# Expression and clinicopathological significance of *Mel-18 *and *Bmi-1 *mRNA in gastric carcinoma

**DOI:** 10.1186/1756-9966-29-143

**Published:** 2010-11-08

**Authors:** You-Wei Lu, Jin Li, Wei-Jian Guo

**Affiliations:** 1 Department of Medical Oncology, Fudan University Shanghai Cancer Center; China; 2 Department of Oncology, Shanghai Medical College, Fudan University, Shanghai 200032, China

## Abstract

**Background:**

The *Polycomb *group (*PcG*) genes are a class of regulators responsible for maintaining homeotic gene expression throughout cell division. *PcG *expression is deregulated in some types of human cancer. Both Bmi-1 and Mel-18 are of the key PcG proteins. We investigate the expression and clinicopathological roles of *Mel-18 *and *Bmi-1 *mRNA in gastric cancer.

**Methods:**

The expression of *Mel-18 *and *Bmi-1 *in a series of 71 gastric cancer tissues and paired normal mucosal tissues distant from the tumorous lesion was assayed by quantitative real time RT-PCR. The correlation between *Mel-18 *and *Bmi-1 *mRNA expression, and between *Mel-18 *or *Bmi-1 *mRNA level and clinicopathological characteristics were analyzed.

**Results:**

Expression of *Mel-18 *and *Bmi-1 *genes was variably detected, but overexpression of *Bmi-1 *mRNA and decreased expression of *Mel-18 *mRNA were the most frequent alteration. In addition, the expression of *Bmi-1 *and *Mel-18 *mRNA inversely correlates in gastric tumors. Moreover, a significant positive correlation between *Bmi-1 *overexpression and tumor size, depth of invasion, or lymph node metastasis, and a significant negative correlation between *Mel-18 *low-expression with lymph node metastasis or the clinical stage were observed.

**Conclusion:**

Our data suggest that *Mel-18 *and *Bmi-1 *may play crucial but opposite roles in gastric cancer. Decreased *Mel-18 *and increased *Bmi-1 *mRNA expression was associated with the carcinogenesis and progression of gastric cancer. It is possible to list *Bmi-1 *and *Mel-18 *as biomarkers for predicting the prognosis of gastric cancer.

## Background

The *Polycomb *group (*PcG*) genes were first identified in Drosophila as a class of regulators responsible for maintaining homeotic gene expression throughout cell division [1], *PcG *genes are conserved from Drosophila to mammals, and the expression levels of mammalian *PcG *genes differ between different tissues and cell types [2], *PcG *genes act as epigenetic silencers during embryo morphogenesis with a central role in the nervous system, heart, and skeleton development [3-7].In addition, *PcG *members have been involved in the regulation of such adult processes as the cell cycle, X-inactivation, and hematopoiesis [8-14]. *PcG *expression is deregulated in some types of human cancer [15].Moreover, several PcG genes may regulate the self-renewal of specific stem cell types, suggesting a link between the maintenance of cell homeostasis and carcinogenesis [16,17].

Bmi-1 is one of the key PcG proteins. It was initially identified as an oncogene that cooperated with c-Myc in the generation of mouse pre-B-cell lymphomas. It is also considered the first functional mammalian *PcG *protooncogene to be recognized, and it has been implicated in axial patterning, hematopoiesis, cell cycle regulation, and senescence [18-21]. Human *Bmi-1 *gene is located at the short arm of chromosome 10p13 [22], The region is involved in chromosomal translocations in leukemia and is amplified in non-Hodgkin's lymphoma as well as in solid tumors [23]. Bmi-1 induces S-phase entry by inhibiting Rb function via repression of the INK4a/ARF locus [24-26]. Moreover, overexpression of *Bmi-1 *in mammary epithelial cells may activate telomerase and lead to immortalization [27]. Overexpression of *Bmi-1 *has been found in several human malignancies including breast cancer, colorectal cancer, nasopharyngeal carcinoma, melanoma, gastric cancer, and bladder cancer [28-33]. Overexpression of *Bmi-1 *often correlates with poorer prognosis and treatment failure [30,32-34]. *Bmi-1 *also plays an important role in self-renewal of hematopoietic stem cells, neural stem cells and mammary stem cells [35-37].

In addition to *Bmi-1*, mammalian cells also express a Bmi-1-related PcG protein Mel-18. The *Mel-18 *gene product is structurally highly similar to Bmi-1 protein. Interestingly, we have found that *Bmi-1 *is negatively regulated by *Mel-18 *and expression of *Mel-18 *negatively correlates with *Bmi-1 *in breast tumors, and *Mel-18 *overexpression in breast cancer cell line MCF7 results in downregulation of *Bmi-1 *and reduction of transformed phenotype [38]. Negative correlation between *Bmi-1 *and *Mel-18 *expression was also recently reported in hematopoietic stem cells [39]. Lee et al. also recently reported that overexpression of *Mel-18 *inhibits growth of breast cancer cells [40]. These data suggested that *Mel-18 *acts as a potential tumor suppressor. However, the function of *Mel-18 *is still debatable. In few other studies, it was found that similar to *Bmi-1, Mel-18 *can act as an oncogene [41,42]. So, the role of *Mel-18 *in cancers other than breast cancers and different pathological conditions is still not clear and need to be clarified.

Gastric cancer is one of the most common malignancies throughout the world. It has been reported that *Bmi-1 *is overexpressed in gastric cancer and is an independent prognosis factor [32]. We have also studied the expression of Mel-18 and Bmi-1 in gastric tumors by immunohistochemistry (IHC). We found that gastric tumor tissues expressed significantly higher Bmi-1 and lower Mel-18, and the expression of Mel-18 negatively correlated with Bmi-1; there was a significant positive correlation between Bmi-1 expression with lymph node metastasis, or clinical stage, but there was no obvious correlation between Mel-18 expression and clinicopathological factors; downregulation of *Bmi-1 *by *Mel-18 *overexpression or knockdown of Bmi-1 expression was accompanied by decreased transformed phenotype and migration ability in gastric cancer cell lines in *in vitro *study[33]. So, the results of Bmi-1 expression correlated with lymph node metastasis or clinical stage in *in vivo *study was accordance with the results in *in vitro *study, while the results of no correlation was found between Mel-18 expression and clinicopathological factors in *in vivo *study was not accordance with the results in *in vitro *study, we suspected that one of the reason may due to the reliability of IHC method which was used to detect the expression of Bmi-1 and Mel-18 in tumor tissues in most paper of literature including our previous study. This method lacks standard procedure and evaluation criterion and its' reliability depends on the specific of antibody. The results of quantitative Real time RT-PCR (QRT-PCR) with specific primer is more reliable than that of IHC to measure the gene expression level especially for Mel-18, which lacks specific mouse monoclonal antibody till now. Here, we examine the expression of *Mel-18 *and *Bmi-1 *at mRNA level by using QRT-PCR method in a series of gastric cancer, and evaluate the correlation between *Mel-18 *and *Bmi-1 *expression levels. Furthermore, a correlation study between expression levels of both the analyzed genes and several clinical pathologic variables of the tumors was designed. In this study, we characterized the expression profile of *Mel-18 *and *Bmi-1*, and their clinical significance in gastric cancer.

## Materials and methods

### Clinical samples

Human gastric cancer samples were obtained from patients who underwent surgery for gastric cancer in our hospital from 2007 to 2008. All of the patients didn't receive prior chemotherapy or radiotherapy before surgery. A total of 71 fresh gastric tissues and paired normal mucosal tissues distant from the tumorous lesion were removed and frozen in liquid nitrogen and stored at -80°C until further use. After the diagnosis of gastric cancer was confirmed, RNA was extracted with Trizol reagent (Invitrogen) according to the manufacturer's protocol from the cancerous and paired normal tissues for further RT-PCR analysis of *Mel-18 *and *Bmi-1 *expression. By pathological types, all cases of gastric cancer are adenocarcinomas.

The clinicopathologic variables were obtained from the medical records and the disease stages of the patients were classified according to the 2002 UICC gastric cancer TNM staging system. Prior patients' consent and approval from the Institute Research Ethics Committee were obtained for the use of clinical materials described in the present study.

### Quantitative real time RT-PCR (QRT-PCR) assays

The QRT-PCR was carried out as described using Brilliant SYBR Green QRT-PCR Master Mix, 2-Step kit (Stratagene, La Jolla, CA) [43]. cDNA was synthesized using reverse transcriptase, and the PCR amplification was carried out using PTC-200 Real Time PCR system (MJ Research Inc, USA). The primers for QRT-PCR were Glyceraldehyde-3-phosphate dehydrogenase (*GAPDH*) forward (F)-5' GCTGAACGGGAAGCTCACTG-3',GAPDH reverse (R)- 5'GTGCTCAGTGTAGCCCAGGA3'; *Bmi-1 *F 5' GCTTCAAGATGGCCGCTTG 3',Bmi-1 R 5'-TTCTCGTTGTTCGATGCATTTC-3'; and *Mel-18 *F 5'- GATGGATGTGCCCAGCAAGT-3', Mel-18 R 5'GGAGCCTTGT CGCTGACTGA-3'. All reactions were done in a 20-μl reaction volume in biplicate. PCR amplification consisted of 10 min of an initial denaturation step at 95°C, followed by 40 cycles of PCR at 95°C for 30 sec, 58°C for 30 sec and 72°C for 30 sec. Standard curves were generated and the relative amount of target gene mRNA was normalized to GAPDH. Specificity was verified by melt curve analysis and agarose gel electrophoresis. Data normalization and analysis an endogenous control, GAPDH present on the PCR was used for normalization. Each replicate cycle threshold (CT) was normalized to the average CT of endogenous control on a sample basis. The comparative CT method was used to calculate the relative quantification of gene expression. The following formula was used to calculate the relative amount of the transcripts in the gastric cancer samples and the control group, both of which were normalized to the endogenous control. ΔΔCT = ΔCT (gastric cancer)- ΔCT (control) for RNA samples. ΔCT is the log2 difference in CT between the target genes and endogenous controls by subtracting the average CT of controls from each replicate. The fold change for each gastric cancer sample relative to the control sample = 2^-ΔΔCT^. When the expression showed a 2-fold increase or decrease compared with normal counterpart tissue, it was considered as an altered expression.

### Statistical analysis

All statistical analyses were done by SPSS 15.0 software package. Two-tailed P value less than 0.05 was considered statistically significant. In the set of RT-PCR analysis of fresh tumors and paired normal tissues, the ratio of *Bmi-1 *and *Mel-18 *mRNA expression was not normally distributed. Hence, the distribution was established by using Log_10_, and geometric averages. The correlation between *Bmi-1 *and *Mel-18 *expression levels was analyzed by the Pearson coefficient test. The correlation between *Bmi-1 *or *Mel-18 *expression and clinicopathologic characteristics was analyzed by ANOVA.

## Results

### Expression of *Bmi-1 *and *Mel-18 *at mRNA level inversely correlates in gastric tumors

**Figure 1:**
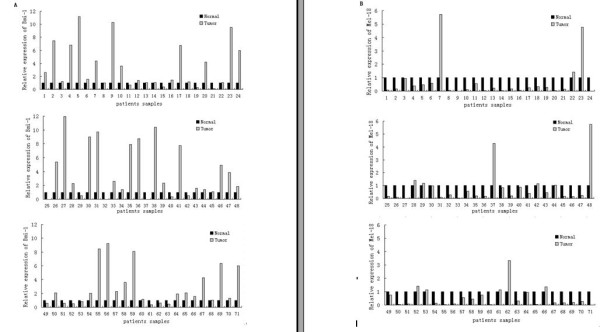
**Comparative expression levels of *Bmi-1 *or *Mel-18 *were shown in 71 normal mucosal tissues and paired gastric cancer samples**. A: *Bmi-1 *gene expression in human gastric cancer. B: *Mel-18 *gene expression in human gastric cancer. Expression level of target genes was displayed in a relative quantification method as a ratio between it in tumor tissues and that in normal tissues in the amounts of RNA. The expression level of *Bmi-1 *or *Mel-18 *in normal tissues was treated as 1 and the ratio of gene expression was the expression level of *Bmi-1 *or *Mel-18 *in tumor tissues.

Our previous data showed an inverse correlation between *Bmi-1 *and *Mel-18 *expression in breast cancer cells and breast cancer tissues. Based on these data, we hypothesized that gastric cancer may also express high *Bmi-1 *and low *Mel-18*. To probe this hypothesis, we studied the expression of *Mel-18 *and *Bmi-1 *in gastric tumors by QRT-PCR. QRT-PCR analysis showed that 35 of 71 (49.3%) fresh gastric tumor tissues overexpressed Bmi-1, and 46 of 71 (64.79%) expressed low levels of Mel-18, compared with paired normal gastric mucosal tissues. (Table 1, Figure 1).

**Table 1:** Frequencies of altered expression of *Bmi-1 *and *Mel-18 *in the 71 gastric cancer tissues

Gene	Decreased expression		Normal expression		Overexpression	
			
	Frequency	Percentage	Frequency	Percentage	Frequency	Percentage
*Bmi-1*	9	12.68%	27	38.03%	35	49.30%
*Mel-18*	46	64.79%	20	28.17%	5	7.04%

The correlation between *Bmi-1 *and *Mel-18 *expression at mRNA level was further analyzed by the Pearson coefficient correlation analysis, which showed a strong negative correlation (r = - 0.252, P = 0.034).

### The correlation between the expression of *Mel-18 *or *Bmi-1 *with clinicopathologic characteristics

We found a significant positive correlation between *Bmi-1 *overexpression and tumor size, depth of invasion (T classification), or lymph node metastasis (N classification). The expression of *Bmi-1 *was higher in the patients with bigger tumor, deeper invasion, or positive lymph node metastasis. We also found that there was a significant negative correlation between *Mel-18 *expression with lymph node metastasis or the clinical stage. Its expression was lower in the patients with lymph node metastasis, or late stage disease (Table 2).

**Table 2:** Correlations between the expression level of *Bmi-1 *or *Mel-18 *and clinical-pathologic variables

Variable	*Bmi-1*			*Mel-18*		
		
	n	GA	P	n	GA	P
Gender						
Male	58	1.568	0.687	58	0.259	0.309
Female	13	1.958		13	0.150	
Age(years)						
<60	44	1.584	0.832	44	0.188	0.166
≥60	27	1.715		27	0.336	
Size (cm)						
<4.5	26	0.965	0.049*	26	0.206	0.335
≥4.5	45	2.213		45	0.313	
Histology						
Moderately differentiated	13	0.989	0.248	13	0.185	0.584
Poorly differentiated	58	1.827		58	0.247	
T classification						
T1/2	12	0.635	0.036*	12	0.399	0.242
T3/4	59	1.979		59	0.210	
LNM						
Negative	16	0.762	0.044*	16	0.513	0.037*
Positive	55	2.038		55	0.186	
Distant metastasis						
Negative	68	1.663	0.597	68	0.232	0.645
Positive	3	2.932		3	0.372	
Clinical Stage						
I/II	22	0.949	0.075	22	0.506	0.010*
III/IV	49	2.084		49	0.166	

Abbreviations: LNM, lymph node metastases; GA, geometrical average; *, Statistically significant. Statistically significant at 0.05 level (bilateral).

## Discussion

Mammalian PcG protein complexes are generally classified into two distinct types: Polycomb repressive complexes 1 and 2 (PRC1 and PRC2). Mel-18 protein product is a constituent of mammalian PRC1 together with M33, Bmi-1 or rae28/Mph-1, and Scmh1 [1,44-47]. In human tumors, some reports have showed alterations in PcG expression, in such human hematologic malignancies as nodal B-cell lymphomas [48,49], mantle cell lymphomas [23,50], and Hodgkin's lymphomas [13,51,52].It has been reported that solid tumors, such as lung cancers [53],medulloblastomas [3], liver [54], penis [55], breast [28,56],colon [57], and prostate carcinomas [58], also display disturbed *PcG *gene expression.

Bmi-1 is one of the most important PcG proteins that is known to regulate proliferation and senescence in mammalian cells, and plays an important role in self-renewal of stem cells. It can not only immortalize human mammary epithelial cells (HMECs) [27], but also can cooperate with H-Ras to transform HMECs and transform keratinocytes [59,60]. Abnormal expression of Bmi-1 has been found in several human cancers and its overexpression is often correlated with poor prognosis in many types of malignances [28-34]. Overexpression of Bmi-1 in gastric cancer has been previously reported[32,61]. It was found that Bmi-1 overexpression was highly correlated with tumor size, clinical stage, lymph node metastasis and T classification [32]. In another study, Bmi-1 expression was closely related with the Lauren's and Borrmann's classification and clinical stage in gastric cancer [61]. We also found that gastric tumor tissues expressed significantly higher Bmi-1, and Bmi-1 overexpression correlated with lymph node metastasis, or clinical stage, which was accordance with the results in *in vitro *study that knockdown of Bmi-1 expression was accompanied by decreased transformed phenotype and migration ability in gastric cancer cell lines [33]. In these studies Bmi-1 was detected at protein level by IHC method. Here we detected *Bmi-1 *at mRNA level by QRT-PCR method and found that *Bmi-1 *is overexpressed in gastric tumors and *Bmi-1 *overexpression correlates with tumor size, depth of invasion (T classification), or lymph node metastasis (N classification), which confirms previous observation of Bmi-1 at protein level. It suggests that *Bmi-1 *may play a crucial role and act as an oncogene in gastric cancer, and associated with the carcinogenesis, progression, and metastasis of gastric cancer.

*Mel-18 *was originally cloned from B16 mouse melanoma cells [62]. *Mel-18 *may bind to the nucleotide sequence 5'-GACTNGACT-3', which is present in the promoter region of certain genes. One of the unique target genes of *Mel-18 *is c-Myc transcriptionally repressed by *Mel-18*. In mature resting B cells, *Mel-18 *negatively regulates B cell receptor-induced proliferation through the down-regulation of the c-Myc/cdc25 cascade [63,64]. Our previous studies suggest that *Mel-18 *is a physiologic regulator of *Bmi-1 *expression and transcriptionally down-regulates *Bmi-1 *expression during senescence in human fibroblasts and acts as a tumor suppressor in breast cancer [38,43]. Our previous data also showed an inverse correlation between Bmi-1 and Mel-18 expression at protein level in breast cancer and gastric cancer [33,38]. However, there was no correlation between Mel-18 expression at protein level and clinicopathological factors in *in vivo *study, which was not accordance with the results in *in vitro *study that Mel-18 overexpression was accompanied by decreased transformed phenotype and migration ability in gastric cancer cell lines[33]. One of the reasons may due to the reliability of IHC method depends on the specific of antibody. Mel-18 antibody is rabbit polyclonal and it's specific is not so good as Bmi-1 antibody which is mouse monoclonal. So we suspect the results of Mel-18 expression in tumor tissues at protein level detected by IHC may be not too reliable. To clarify this problem and further explore the role of Mel-18 in gastric cancer, we detected it's expression at mRNA level by QRT-PCR in the present study. We found that most gastric tumor tissues (64.79%) expressed decreased mRNA levels of *Mel-18*, and there was a strong negative correlation between *Bmi-1 *and *Mel-18 *expression at mRNA level. The results confirm the expression of Mel-18 and its' relationship with Bmi-1 at protein level in our previous study. More important, we also found that decreased expression of *Mel-18 *correlated with lymph node metastasis or the clinical stage, which was accordance with the results in *in vitro *study that *Mel-18 *overexpression was accompanied by decreased transformed phenotype and migration ability in gastric cancer cell lines in our previous study[33]. It provides more convincing *in vivo *data to suggest that *Mel-18 *may play a crucial opposite role to *Bmi-1 *and act as a tumor suppressor in gastric cancer, and associated with the carcinogenesis, progression, and metastasis of gastric cancer.

In the current study we demonstrated that neoplastic cells in gastric cancer can't normally express *Bmi-1 *and *Mel-18*. We propose that abnormal *PcG *expression results in an altered composition of the PRC1 in gastric cancer cells, which probably affects expression of target genes involved in regulation of senescence and/or the cell cycle. Our observations add to the increasing evidence that *PcG *genes are very important contributors to the carcinogenesis and progression of human tumors. We additonally found that both *Mel-18 and Bmi-1 *correlated with lymph node metastasis. The mechanisms that they regulate cancer cells metastasis need to be further studied.

This research is the first time to study the correlation between *Mel-18 *or *Bmi-1 *expression at mRNA level and clinicopathological characteristics of gastric cancer by quantitative method. The expression of *Bmi-1 *and *Mel-18 *was correlated with gastric cancer progress, advanced gastric cancer more likely expressed higher *Bmi-1 *and lower *Mel-18*. Its clinical value deserves further study in a larger patient population.

## Conclusions

In conclusion, our results suggest that *Bmi-1 *and *Mel-18 *are coordinately deregulated. Interestingly, we observed a reverse correlation between the expression levels of *Bmi-1 *and *Mel-18 *in gastric cancer. Both *Bmi-1 *and *Mel-18 *are involved in the development and progression of gastric cancer. *Bmi-1 *and *Mel-18 *might be novel molecular markers for gastric cancer. But,the detailed mechanisms of regulation of *Bmi-1 *and *Mel-18 *remained to be elucidated.

## Competing interests

The authors declare that they have no competing interests.

## Authors' contributions

LYW performed the experiment and prepared the manuscript; LJ supervised the experiment; GWJ designed the experiment and supervised the project. All authors have read and approved the final manuscript.
